# Shiga Toxin–producing *Escherichia coli* Strains Negative for Locus of Enterocyte Effacement

**DOI:** 10.3201/eid1502.080631

**Published:** 2009-03

**Authors:** Hayley J. Newton, Joan Sloan, Dieter M. Bulach, Torsten Seemann, Cody C. Allison, Marija Tauschek, Roy M. Robins-Browne, James C. Paton, Thomas S. Whittam, Adrienne W. Paton, Elizabeth L. Hartland

**Affiliations:** Monash University, Melbourne, Victoria, Australia (H.J. Newton, J. Sloan, D.M. Bulach, T. Seemann, C.C. Allison, E.L. Hartland); University of Melbourne, Melbourne (H.J. Newton, J. Sloan, M. Tauschek, R.M. Robins-Browne, E.L. Hartland); University of Adelaide, Adelaide, South Australia, Australia (J.C. Paton, A.W. Paton); Michigan State University, East Lansing, Michigan, USA (T.S. Whittam); 1 These authors contributed equally to this article.

**Keywords:** STEC, molecular evolution, EhxA protein, Escherichia coli plasmids, foodborne pathogens, research

## Abstract

Most Shiga toxin–producing *Escherichia coli* (STEC) infections that are associated with severe sequelae such as hemolytic uremic syndrome (HUS) are caused by attaching and effacing pathogens that carry the locus of enterocyte effacement (LEE). However, a proportion of STEC isolates that do not carry LEE have been associated with HUS. To clarify the emergence of LEE-negative STEC, we compared the genetic composition of the virulence plasmids pO113 and pO157 from LEE-negative and LEE-positive STEC, respectively. The complete nucleotide sequence of pO113 showed that several plasmid genes were shared by STEC O157:H7. In addition, allelic profiling of the *ehxA* gene demonstrated that pO113 belongs to a different evolutionary lineage than pO157 and that the virulence plasmids of LEE-negative STEC strains were highly related. In contrast, multilocus sequence typing of 17 LEE-negative STEC isolates showed several clonal groups, suggesting that pathogenic LEE-negative STEC has emerged several times throughout its evolution.

## Abstract 2

The *ehx* plasmids of these strains are highly related, which suggests acquisition of the large plasmid was central to the strains’ emergence.

Shiga toxin–producing *Escherichia coli* (STEC) strains are foodborne enteric pathogens associated with hemorrhagic colitis and the development of the life-threatening condition hemolytic uremic syndrome (HUS). Young children in industrialized countries are particularly at risk (*1*). The production of Shiga toxin (Stx) increases mortality rates for those persons who have STEC infections compared with those who have other varieties of *E. coli* infection (*1*). Therefore, the production of >1 Stx variants is central to pathogenesis; however, bacterial adherence and subsequent colonization of the intestinal epithelium also contribute to STEC virulence.

Many disease-related STEC serogroups, including the most prevalent O157:H7 clone, possess a chromosomal pathogenicity island termed the locus of enterocyte effacement (LEE) (*2*). STEC containing LEE are characterized by their ability to attach to the host intestinal mucosa and destroy the surrounding microvillus brush border, which causes substantial cytoskeletal rearrangements within the enterocyte (*3*). On the basis of this phenotype, LEE-positive STEC are classified as attaching and effacing (A/E) pathogens with the closely related human pathogen, enteropathogenic *E. coli* (*4*,*5*). Although many studies have demonstrated that LEE is essential for host colonization and virulence of A/E pathogens (*6*–*9*), others have demonstrated that some STEC isolates without LEE, such as STEC O113:H21, are associated with sporadic and outbreak cases of severe disease indistinguishable from that caused by STEC O157:H7 (*10*–*13*). In the absence of LEE, mechanisms are emerging by which these atypical or LEE-negative STEC interact with the host intestinal mucosa and induce disease. Recently, a potent new toxin, SubAB, was discovered in LEE-negative strains of STEC; this toxin induces cell death through cleavage of the endoplasmic reticulum chaperone, BiP/GRP78 (*14*). SubAB is more prevalent in LEE-negative than in LEE-positive strains of STEC and likely contributes to the progression to severe disease (*15*,*16*). In addition, LEE-negative STEC isolates from several serogroups, particularly STEC O113:H21, can invade tissue culture cells, a mechanism partially dependent on flagellin (*17*–*19*).

Large plasmids encoding EHEC hemolysin (Ehx) are found in almost all disease-associated STEC strains. Restriction fragment length polymorphism (RFLP) analysis of the *ehxA* gene from pO113 of STEC O113:H21 has suggested that pO113 evolved separately to the Ehx plasmids of LEE-positive STEC (*20*). To clarify the contribution of large plasmids to the virulence and evolution of STEC, we determined the complete nucleotide sequence of pO113 from EH41, a clinical (HUS) isolate of STEC O113:H21 (*21*). We compared the nucleotide sequences of pO113 and pO157 to examine the relationship between the 2 plasmids for their origin, gene content, and putative role in disease. In addition, we performed allelic profiling of *ehxA* and *repA* (a plasmid replication initiation gene of pO113) from LEE-negative and LEE-positive STEC isolates to model plasmid evolution compared with the evolution of the *E. coli* background, which was determined by multilocus sequence typing (MLST). Finally, to increase understanding of the evolutionary origins of STEC, we determined genetic features of LEE-negative STEC that may be used to improve diagnosis and detection.

## Materials and Methods

### Bacterial Strains and Culture Conditions

*E. coli* isolates used in this study are listed in Table 1; those shown in boldface were further examined for detailed analysis of *ehxA* and *repA* sequence and MLST. All *E. coli* strains were cultured aerobically in Luria broth or on Luria broth agar at 37°C. When required, chloramphenicol was added at a concentration of 12.5 μg/mL.

**Table 1:** *Escherichia coli* isolates used in this study*

Isolate	Serogroup	Origin†	LEE‡	Ref.
**EH41**	O113:H21	HUS	–	(*17*,*21*,*22*)
**EH53**	O113:H21	HUS	–	(*11*,*23*)
**EH71**	O113:H21	HUS	–	(*17*)
**97025659**	O113:H21	TTP	–	
**95016910**	O113:H21	Food	–	
**95063160**	O113:H21	Cow	–	
95063151	O113:H21	Human	–	
96037512	O113:H21	Food	–	
97001061	O113:H21	Food	–	
99008358	O113:H21	Dysentery	–	
**EH42**	O116:H21	HUS	–	(*17*)
**EH43**	O130:H11	HUS	–	(*11*)
**EH48**	O5:H–	HUS (UTI)	–	
**9724772**	O5:H–	Diarrhea	–	
**EH69**	O1:H7	HUS	–	(*11*)
**EH52**	NT:H7	HUS	–	(*11*,*23*)
**9816261**	O76:H7	HUS	–	
**9611588**	O128:H2	Diarrhea	–	
**EH5**	O91:H–	Diarrhea	–	(*17*)
**EH32**	O91:H–		–	
9730196	O87:H16	Asymptomatic	–	
**9619262-1**	OR:H–	Diarrhea	–	
96/4591	O123:H–	Cow	–	
85-170	O157:H7	HUS	+	
EDL933	O157:H7	HUS	+	(*24*)
84-284	O157:H7		+	
EH9	O157:H7		+	
**9515477**	O157:H7	HC	+	
**9515480**	O157:H7	HUS	+	
**9515474**	O157:H7	HUS	+	
9924822	O157:H7	HUS	+	
**95005698**	O157:H–	HUS	+	
**95051613**	O157:H–	HC	+	
**EH70**	O157:H–	HC	+	
**E45035**	O111:H–	HUS	+	
**ED142**	O111:H–	HUS	+	
**EH38**	O111:H–	HUS	+	
**EH44**	O26	HUS	+	
**EH6**	O26:H11		+	
EH34	O26:H11		+	
**EH1**	O26:H21	Diarrhea	+	
EH68	O147:H–	Diarrhea	+	
**EH22**	O145:H25		+	

*Isolates shown in **boldface** were used for allelic profiling and multilocus sequence typing phylogenetic analysis. LEE, locus of enterocyte effacement; Ref., reference; HUS, hemolytic uremic syndrome; TTP, thrombocytopenic purpura; UTI, urinary tract infection; HC, hemorrhagic colitis. †Clinical information and source are provided where known. ‡Symbols indicate presence (+) or absence (–) of the *eae* gene.

### Sequencing and Annotation of pO113

The complete nucleotide sequence of pO113 from EH41 was determined from a series of overlapping 30–40 kb fragments cloned into the Copy Control pCC1FOS cosmid vector (Epicentre, Madison, WI, USA) and propagated in *E. coli* EPI300 (Epicentre). The final sequence was assembled using Sequencher version 4.7 (Gene Codes Corp. Ann Arbor, MI, USA). The sequence annotation was performed by using WASABI (*25*), a Web-based annotation system for prokaryotic organisms. WASABI was used to generate an automatic annotation of the sequence, which was followed by manual curation by the authors. The automatic annotation used GeneMarkS (http://exon.gatech.edu/genemark) to identify putative coding regions and BLAST (www.ncbi.nlm.nih.gov/blast) and reversed position-specific (RPS)–BLAST to assign function on the basis of sequence similarity. The final annotated sequence was deposited into GenBank under accession no. AY258503.

### Presence of pO113 Genes in STEC Strains

DNA for PCR and sequencing was extracted from 1 mL of overnight culture by using the DNeasy Blood and Tissue Kit (QIAGEN, Hilden, Germany). PCR was used to examine the prevalence of 9 genes found on pO113 in a cohort of LEE-negative and LEE-positive STEC strains. The plasmid locations of *pilQ*, *epeA,* *trbC, repA, ehxA, espP, iha, subAB,* and *repZ* are listed in Table 2; oligonucleotide sequences and annealing temperatures were used to amplify fragments of these genes with Taq DNA polymerase (Roche Diagnostics, Mannheim, Germany).

**Table 2:** Gene names, plasmid location, and oligonucleotide sequences used to examine the prevalence of pO113-encoded genes among STEC strains*

Gene	Plasmid location, bp	Oligonucleotide sequences	Amplicon size, bp	PCR annealing temperature, °C
*pilQ*	9593–12105	F: 5’-TTGCAGACCCGCAGTTG-3’ R: 3’-CAGGGCTTCGGCGATGT-5’	870	52
*epeA*	48716–52795	F:5’-CAGGTGGTACTGTCGGC-3’ R: 3’-GCCCATGCCGCTCTGAA-5’	667	46
*trbC*	57357–59660	F: 5’-GCCACCACCGGTGGCGG-3’ R: 3’-CAATCAGAATGCGGTCG-5’	230	50
*repA*	106112–107089	F: 5’-AAAGTCTTGTATAGCTC-3’ R: 3’-GTTATCCATATCCAGGC-5’	871	44
*ehxA*	114136–117132	F: 5’-CCCAGGAGAAGAAGTCA-3’ R: 3’-CTTCACCTGAGGCATCTT-5’	1,108	48
*espP*	134205–140707	F: 5’-AAACAGCAGGCACTTGAACG-3’ R: 3’-GGAGTCGTCAGTCAGTAGAT-5’	2,000	52
*iha*	146066–149549	F: 5’-TCCAGTCAGTACCACGA-3’ R: 3’-CTGTCGGAAAGTTTCAC-5’	981	48
*subAB*	150678–152419	F: 5’-GTGTACAGGACTCATGG-3’ R: 3’-ATCACCAGTCCACTCAG-5’	783	48
*repZ*	163918–164949	F: 5’-ATACAGGAGTAAAACCG-3’ R: 3’-CATATAACGCAGTACAC-5’	1,792	46

*STEC, Shiga toxin–producing *Escherichia coli*; F, forward primer; R, reverse primer.

### Phylogenetic Analysis and MLST

A neighbor-joining tree was created from the nucleotide sequence of the 1,108-bp amplicon of *ehxA* and the 871-bp amplicon of *repA* for 30 *E. coli* isolates. The tree was inferred by using the neighbor-joining method as implemented in ClustalW (*26**,**27*). Significant nodes were identified by bootstrapping and Monte Carlo randomization; nodes present in >70% of the 1,000 bootstrap trees were identified as significant. MLST was performed on the basis of the nucleotide sequence of 580- to 672-bp amplicons of 7 conserved housekeeping genes: *aspC*, *clpX*, *fadD*, *icdA*, *lysP*, *mdh*, and *uidA*. A detailed protocol of the MLST procedure, including allelic type and sequence type (ST) assignment methods, can be found at the EcMLST Web site (www.shigatox.net/mlst). Sequences were concatenated for phylogenetic analyses. A neighbor-joining tree was constructed by using the Kimura 2-parameter model of nucleotide substitution with MEGA3 software (*28*), and the inferred phylogenies were each tested with 1,000 bootstrap replications.

## Results

### Sequence of the Large Plasmid pO113

**Figure 1:**
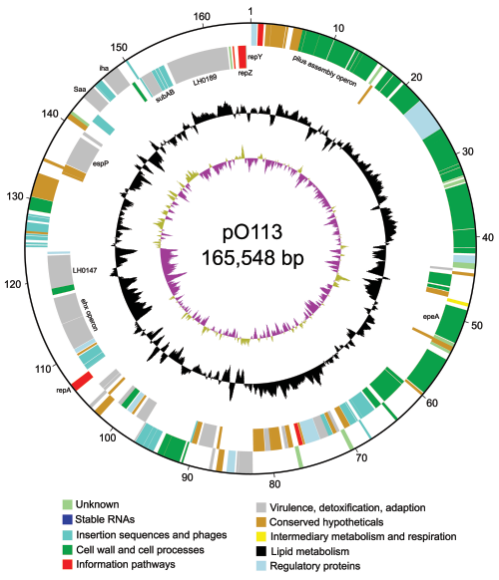
Circular map of virulence plasmid pO113 generated by using circular_diagram.pl (Sanger Institute, Cambridge, UK) and Inkscape software (www.inkscape.org). The locations of proteins encoded on the leading and lagging strand are shown on the outer 2 rings. The colors indicate the assigned GenoList functional category (Institut Pasteur, Paris, France). The black ring indicates GC content, with high GC content outermost. The innermost ring shows GC skew.

**Figure 2:**
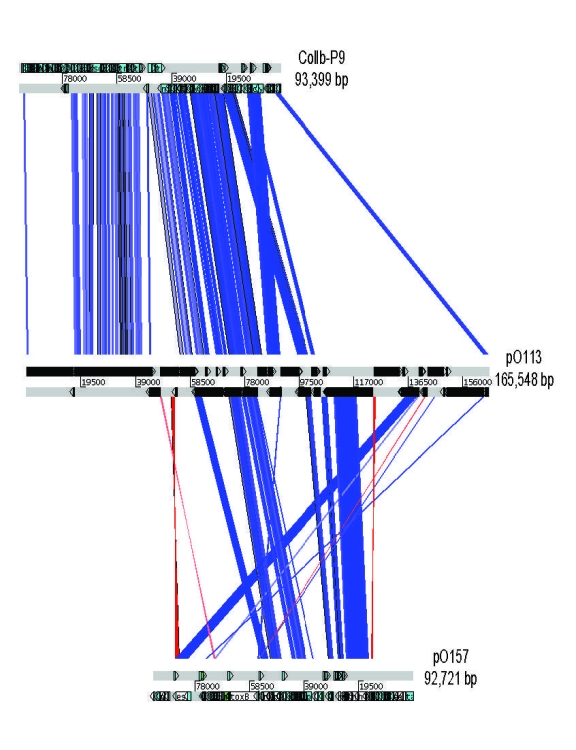
Graphic overview of sequences related to virulence plasmid pO113 in the plasmids ColIb-P9 and pO157. The overview was generated by ACT (www.sanger.ac.uk); related sequences are indicated as boxes between the horizontal bars representing each of the plasmid sequences. Similarity between sequences was established by using TBLASTX with the pO113 sequence as the subject and either ColIb-P9 or pO157 as the query sequence. Blue indicates that open reading frames occur in the same order; red indicates a DNA inversion.

The entire sequence of pO113 comprised 165,548 bases with an overall GC content of 49.64%; the sequence was predicted to encode 155 genes and 38 pseudogenes (Figure 1). Plasmid pO113 was considerably larger than the 92-kb pO157 because of the presence of a 63.9-kb transfer region (*21**,**29*). This region showed similarity to the *tra/trb* regions of the self-transmissible IncI plasmids R64 and ColIb-P9 (307); the number and order of genes within this region were relatively uninterrupted (Figure 2). Most genes acquired by pO113 encoding putative virulence determinants appear to have accumulated outside the transfer region (Figure 2), and many were associated with predicted insertion elements and DNA recombinases (Figure 1). Several pO113-encoded genes were shared by STEC O157:H7, including *ehxCABD* (110,523–117,649 bp), *espP* (135,505–139,407 bp)*,* the putative adhesin *iha* (146,764–148,851 bp), and 2 replication genes, *repA* (106,112–107,089 bp) and *repZ* (163,918–164,949 bp) (*20*). However, pO113 lacked the pO157-encoded type II secretion system and a homologue of the adherence-promoting protein ToxB (*31*).

Plasmid pO113 encoded a number of unique virulence-associated determinants, including the autotransporter protein EpeA (48,716–52,795 bp), the autoagglutinating adhesin Saa (143,552–145,156 bp), and the subtilase-like serine protease toxin SubAB (151,027–152,070) (*15*). In addition, the complete nucleotide sequence of pO113 showed several novel putative factors that have not been described previously and that may contribute to host-pathogen interactions. These factors included the putative product of *LH0147* (118,905–123,200 bp), which may represent a novel member of the trimeric autotransporter family of exported adhesins (*32*). *LH0147* has a putative signal peptide cleavage site between amino acids 15 and 16 and a predicted location in the outer membrane. Similar to Saa, the predicted product of *LH0147* carries a YadA-like, Hia-like, C-terminal region denoted by the conserved domain pfam03895, which may be important for oligomerization and targeting of the protein to the outer membrane (*32**,**33*). In addition, *LH0189* (154,379–162,484 bp) encodes a putative hemolysin/hemagglutinin-like protein that shows significant similarity to several members of the ShlA/HecA/FhaA family of large outer membrane adhesins, including HecA from *Erwinia chrysanthemi* (49% similarity over 1,389/2,801 amino acids) (*34*). However, unlike other members of the HecA family, the product of *LH0189* did not have a recognizable signal peptide sequence for export, although the presence of >2 predicted transmembrane domains suggested that the protein localizes to the bacterial inner membrane. A partial 36,841-bp sequence for pO113 from STEC O113:H21 strain 98KN2 is available from GenBank under accession no. AF399919.3. A comparison of the overlapping regions of both plasmids showed that the nucleotide sequence was highly conserved and that the location, order, and predicted amino acid sequences of putative open reading frames were also highly conserved, exhibiting 99% identity. Although the putative HecA-like adhesin encoded by *LH0189* was apparently absent from STEC O113:H21 strain 98NK2, subsequent PCR analysis by using the primers 5′-TGA TAT TCT GTT GAG TG-3′ and 5′-ATC CGC CAC CTG ACT GC-3′ showed that this gene was also present in STEC O113:H21 98KN2 (data not shown). *LH0189* may be located in another region of the plasmid or on an island elsewhere in the genome of STEC O113:H21 98NK2.

### Prevalence of pO113 Genes among LEE-negative and LEE-positive STEC

To investigate the prevalence of the pO113 encoded genes *pilQ, epeA, trbC, repA, ehxA, espP, iha, subAB,* and *repZ* among a range of LEE-negative and LEE-positive STEC isolates, we screened our collection of STEC isolates by using PCR. PCR conditions and oligonucleotide sequences are listed in Table 2. Of the 23 LEE-negative strains examined, only 10 (44%) were positive for all 9 genes (Table 3). The replication initiation gene, *repA*, was found in all LEE-negative strains tested, *espP* was present in 17 strains (74%), and *subAB* and *repZ* were each present in 18 strains (78%). Of the 20 LEE-positive STEC isolates examined, *ehxA* was present in 18 strains (90%) and *repA* in 19 strains (95%) (Table 4). In contrast, all LEE-positive STEC strains were negative for *epeA,* and only EH1, serogroup O26:H21, was positive for *subAB*.

**Table 3:** Prevalence of selected pO113 ORFs among LEE-negative strains of STEC*

Strain	Gene								
	*pilQ*	*epeA*	*trbC*	*repA*	*ehxA*	*espP*	*iha*	*subAB*	*repZ*
EH41	+	+	+	+	+	+	+	+	+
EH53	+	+	+	+	+	+	+	+	+
EH71	+	+	+	+	+	+	+	+	+
97025659	+	−	+	+	+	+	+	+	+
95016910	+	+	+	+	+	+	+	+	+
95063160	+	+	+	+	+	+	+	+	+
95063151	+	+	+	+	+	+	+	+	+
96037512	+	−	+	+	+	+	+	+	+
97001061	+	+	+	+	+	+	+	−	+
99008358	+	+	+	+	+	+	+	+	+
EH42	+	+	+	+	+	+	+	+	+
EH43	+	+	+	+	+	+	+	+	+
EH48	+	−	+	+	+	−	+	+	−
9724772	+	−	+	+	+	−	+	+	−
EH69	+	+	+	+	+	+	+	−	+
EH52	+	+	+	+	+	+	+	+	+
9816261	+	+	+	+	+	+	−	−	+
9611588	−	−	−	+	+	−	+	+	−
EH5	−	−	−	+	+	+	+	−	−
EH32	−	−	+	+	+	−	+	+	+
9730196	−	−	+	+	−	+	+	−	−
9619262-1	+	−	+	+	+	−	+	+	+
96/4591	+	−	+	+	−	−	+	+	+
% Positive	83	57	91	100	91	74	96	78	78

*ORF, open reading frame; LEE, locus of enterocyte effacement; STEC, Shiga toxin–producing *Escherichia coli.*

**Table 4:** Prevalence of selected pO113 ORFs among LEE-positive strains of STEC*

Strain	Gene								
	*pilQ*	*epeA*	*trbC*	*repA*	*ehxA*	*espP*	*iha*	*subAB*	*repZ*
85-170	−	−	−	+	+	+	+	−	−
EDL933	−	−	−	+	+	+	+	−	−
84-284	−	−	−	+	+	+	−	−	−
EH9	−	−	−	+	+	+	+	−	−
9515477	−	−	−	+	+	+	−	−	+
9515480	−	−	−	+	+	+	+	−	−
9515474	−	−	−	+	+	+	+	−	−
9924822	+	−	+	+	+	+	+	−	+
95005698	−	−	−	+	+	+	−	−	−
95051613	−	−	−	+	+	+	+	−	−
EH70	−	−	−	+	+	+	+	−	−
E45035	+	−	+	+	+	−	+	−	−
ED142	+	−	+	+	+	−	+	−	−
EH38	+	−	−	+	+	+	+	−	−
EH44	−	−	−	+	+	+	+	−	+
EH6	+	−	−	+	+	+	+	−	−
EH34	−	−	−	+	−	−	−	−	−
EH1	+	−	+	−	+	+	+	+	+
EH68	−	−	−	+	−	−	+	−	−
EH22	+	−	−	+	+	−	−	−	−
% Positive	35	0	20	95	90	75	75	0.05	20

*ORF, open reading frame; LEE, locus of enterocyte effacement; STEC, Shiga toxin–producing *Escherichia coli.*

### Phylogenetic Analysis of *ehxA* and *repA*

**Figure 3:**
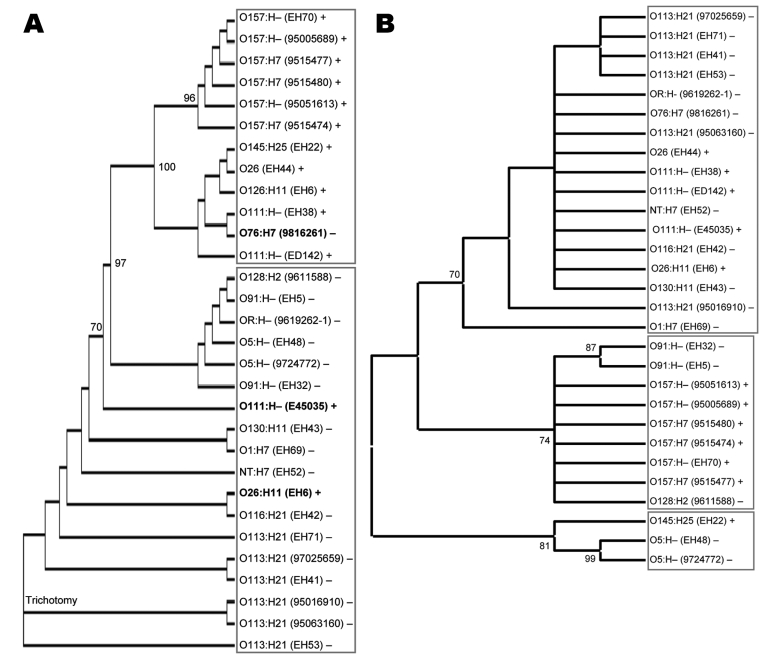
Neighbor-joining tree of *ehxA* (A) and *repA* (B) as implemented in ClustalW (www.ebi.ac.uk/Tools/clustalw2). This rectangular cladogram demonstrates the distinct clades (shown by boxes) for *ehxA* that delineate locus of enterocyte effacement (LEE)–negative and LEE-positive Shiga toxin–producing *Escherichia coli* strains. Exceptions to this pattern are shown in **boldface**, strain names are shown in parentheses, and + or – indicates the presence or absence of LEE. Significant nodes were identified by bootstrapping (Monte Carlo randomization); nodes were present in >70% of the 1,000 bootstrap trees highlighted and identified as significant.

To clarify the genetic relationship and origin of the large plasmids of LEE-negative STEC, we initially performed allelic profiling of the *ehxA* gene. The 1,108-bp amplicon of *ehxA* was sequenced for 17 LEE-negative and 13 LEE-positive STEC strains (Table 1). Overall, the nucleotide sequences of *ehxA* were closely related, exhibiting 96.8% nucleotide sequence identity across all 30 isolates. The nucleotide sequences were analyzed by using ClustalW (www.ebi.ac.uk/Tools/clustalw2) to produce a neighbor-joining tree demonstrating sequence relationships (Figure 3, panel A). The *ehxA* sequences from LEE-negative and LEE-positive isolates segregated into 2 distinct clades, supporting a previous study that suggested pO113 belonged to distinct evolutionary lineage from pO157 (*20*). Irrespective of serotype, here we could show that the LEE-negative Ehx-encoding plasmids were genetically related, which suggests a common evolutionary origin (Figure 3, panel A). However, our study highlighted 3 exceptions to this delineation, including the LEE-negative STEC strain 9816261 (O76:H7), which possesses an *ehxA* sequence most closely linked to O111:H– strains of LEE-positive STEC, and the LEE-positive strains E45035 (O111:H–) and EH6 (O26:H11), which segregated with LEE-negative isolates.

To determine if the phylogenetic relationship between the LEE-positive and LEE-negative plasmids demonstrated by *ehxA* was evident in other pO113-encoded factors, we constructed a neighbor-joining tree for a second pO113 gene, the plasmid replication initiation gene *repA.* The 871-bp amplicon of *repA* was sequenced for 16 LEE-negative and 13 LEE-positive STEC strains. Similar to *ehxA*, *repA* was chosen because it was found in most LEE-negative and LEE-positive STEC strains. Overall, the nucleotide sequences of *repA* were closely related, exhibiting 94.5% nucleotide sequence identity. Phylogenetic analysis of the *repA* nucleotide sequence did not show the same clustering of strains as *ehxA* and showed less distinct delineation on the basis of LEE-positivity (Figure 3, panel B). These findings may indicate that *repA* was acquired by the large STEC plasmids independently of *ehxA* and suggests that *ehxA* is a more discriminating phylogenetic marker for allelic profiling.

### Genetic Diversity and Ancestral Relationship between LEE-negative and LEE-positive STEC

**Figure 4:**
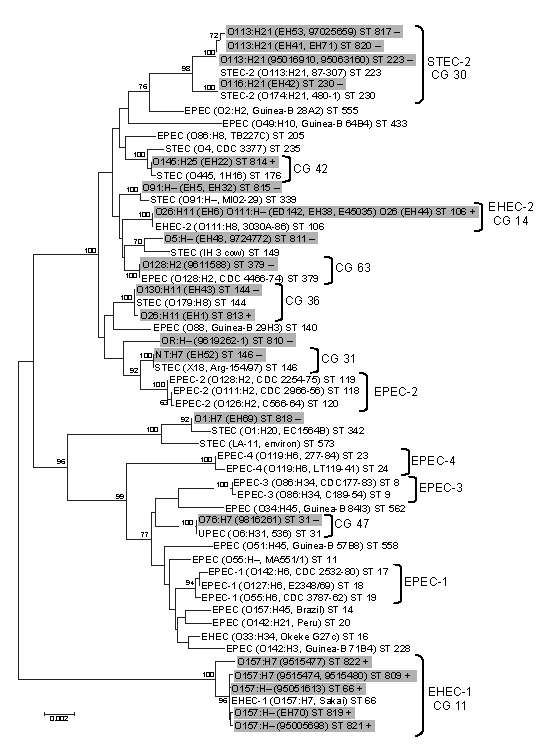
Phylogenetic relationships of 17 locus of enterocyte effacement (LEE)–negative and 13 LEE-positive Shiga toxin–producing *Escherichia coli* (STEC) strains (highlighted in gray) compared with a cohort of reference *E.* strains. Phylogeny was demonstrated by a neighbor-joining algorithm from 7 housekeeping gene sequences. Each isolate has been assigned a sequence type (ST) (in **boldface**), and assigned clonal groups (CGs) are displayed. The scale bar demonstrates the branch length that corresponds to 2 nucleotide substitutions per 1,000 nucleotide sites. Significant nodes were identified by bootstrapping (Monte Carlo randomization); nodes were present in >70% of the 1,000 bootstrap trees highlighted and identified as significant.

Because the large plasmids of LEE-negative STEC appeared to be highly related, we examined the *E. coli* background of these strains by using MLST to understand if the strains also exhibited a clonal relationship. PCR amplification and sequencing of the 7 MLST loci in 17 LEE-negative and 13 LEE-positive STEC strains allowed ST classification of each strain (www.shigatox.net/mlst). The STs of the 30 STEC strains examined were intercalated with previously characterized pathogenic *E. coli* isolates, which showed that the current strains grouped into 7 known clonal groups (CGs) (Figure 4). Of the LEE-positive isolates, 6 belonged to CG 11 or the EHEC-1 group; 5 belonged to CG 14 or EHEC-2. One isolate EH22 (O145:H22), belonged to CG 42, and 1 isolate, EH1 (O26:H11), belonged to CG 36 and contained one of the LEE-negative isolates, EH43 (O130:H11). Among the other LEE-negative isolates examined, 7 belonged to CG 30 or the STEC-2 group, including all the O113:H21 isolates. One isolate, EH52 (NT:H7), belonged to CG 31, and 1 isolate, 9816261 (O76:H7), belonged to CG 47. Isolate 9816261 was most closely related to uropathogenic *E. coli*. Isolate 9611588 (O128:H2) was classified as a member of a new CG, CG 63, together with an enteropathogenic *E. coli* isolate (ST379). The remaining 6 LEE-negative STEC isolates examined here could not be assigned into CGs. These were 2 O5:H– isolates assigned ST811 (isolates 9724772 and EH48), 2 O91:H– isolates assigned ST814 (isolates EH32 and EH5), 1 O1:H7 isolate assigned ST818 (isolate EH69), and 1 OR:H– isolate assigned ST810 (isolate 9619262). Overall, the MLST data showed that LEE-negative STEC do not exhibit clonality, and the large Ehx plasmid was acquired by horizontal gene transfer.

## Discussion

We constructed a complete nucleotide sequence for a large plasmid from LEE-negative STEC. Previous studies have shown that many open reading frames on the plasmid are shared by other STEC, predominantly LEE-negative strains (*15*,*21*,*29*,*33*). Our PCR screen of 17 LEE-negative STEC isolates demonstrated a high degree of conservation of pO113-encoded genes, although not all genes examined were present in all strains. In addition, we found that although *repA*, *ehxA, espP,* and *iha* were common to LEE-positive and LEE-negative STEC, other pO113-encoded genes, *pilQ, epeA, trbC, subAB,* and *repZ*, were more commonly associated with LEE-negative STEC.

Generally, pO113 exhibited a mosaic structure similar to pO157, encoding a large number of transposases and insertion elements clustered outside a plasmid transfer region. Although the remnant transfer region places pO157 in the F-family of conjugative plasmids, the functional transfer region of pO113 places it within the IncI group of self-transmissible plasmids, which includes R64 and ColIb-P9. This finding indicates that pO113 and pO157 have a different evolutionary history. Allelic profiling of the *ehxA* gene in diverse STEC isolates confirmed that the large plasmids belong to different evolutionary lineages, independent of serotype.

In addition to the previously characterized virulence-associated determinants EpeA, Saa, and SubAB (*16*,*21*,*33*), a comparison of the coding capacity of pO113 with pO157 showed that pO113 encoded an array of predicted adhesins and toxins that may contribute to host colonization and disease. Some of the pO113-specific genes have homologs present on the chromosome of STEC O157:H7, which suggests that they may have a common role in STEC pathogenesis and may represent an adaptation to human infection. The unique virulence genes encoded by pO113 (especially those encoding putative adhesins and toxins) may compensate for the lack of A/E lesion formation by STEC O113:H21 and may account for the high virulence of this clone. Although the exact contribution of many of these novel and unique pO113-encoded factors to the pathogenesis of STEC infections in humans remains to be determined, the presence of several genetic determinants shared between pO113 and pO157 suggests that pO113 may contribute to STEC pathogenesis in a manner analogous to pO157. In addition, given the different evolutionary origins of pO157 and pO113, the presence of similar genes in both plasmids suggests that several types of gene transfer have occurred, including transposition, phage insertion, and recombination. Indeed, in pO113 the *ehx* operon, *repA*, *espP,* and *iha* are all closely associated with remnant transposases.

MLST is commonly used to examine the relationship between *E. coli* lineages. Previous MLST studies have used examples of parallel evolution in *E. coli* clones to show that the high virulence of particular clones is not an ancestral state derived from primordial *E. coli* but rather a condition developed from parallel acquisition of bacteriophage-encoded and plasmid-borne virulence determinants (*35*). One such example of parallel evolution is seen in CGs EHEC 1 and EHEC 2. These CGs are characterized by the distinct insertion sites of the LEE pathogenicity island and different intimin subtypes (*35*). On the basis of the *ehxA* sequence, 2 LEE-positive EHEC-2 clones (isolates E45035 and EH6) did not branch according to their LEE profile, which showed a closer relationship to the LEE-negative *ehxA* sequences. This finding suggests that some LEE-positive STEC strains have acquired LEE-negative plasmids. In addition, LEE-negative O76:H7 (isolate 9816261) was more closely related to LEE-positive *ehxA* sequences. MLST demonstrated that the isolate 9816261 represented an unusual STEC ST belonging to CG 47, showing the closest evolutionary link to UPEC.

Phylogenetic analysis of a second pO113 and pO157 gene, *repA*, did not lead to the same clustering of strains as *ehxA*. The 3 clades shown in the *repA* sequence showed less distinct delineation on the basis of the presence or absence of LEE. This disagreement may result because *repA* is not a virulence factor and, therefore, is not subject to the same selective pressure as virulence determinants, such as *ehxA*. Moreover, in pO113 and pO157, *repA* is located next to remnant transposases, and the *repA* sequence may reflect the mosaic structure of the large plasmids rather than their ancestral genetic origin. In addition, we cannot be certain that all the *repA* sequences examined here were located on Ehx-encoding plasmids. Indeed, 2 LEE-positive STEC strains in our study, O26:H11 (EH34) and O147:H– (EH68), were positive for *repA* but not *ehxA,* indicating that *repA* may be present without *ehxA*. In this context, *ehxA* would appear to be a more useful and reliable marker for allelic profiling of the large plasmids.

A previous study that examined the evolution of 56 LEE-negative O174 STEC isolates demonstrated that they fell into 4 separate evolutionary clusters (*36*). Similarly, our study of LEE-negative STECs of various serogroups demonstrated divergent evolution; several clonal groups were represented and 6 other STs remain unassigned. STEC O113:H21 (strain EH41), from which we established the sequence of pO113, is a member of CG 30 or the STEC2 group and is closely related to other O113:H21 isolates by MLST and *ehxA* profiling, suggesting that STEC O113:H21 strains are clonal. The PCR screen that was conducted to examine the presence of pO113 genes in different STEC-2 isolates supports this idea with all but 1 of the O113:H21 strains possessing all 9 of the pO113 genes examined. The study of serogroup O174 isolates also demonstrated that the virulence gene content of these evolutionarily divergent strains is similar, confirming the idea that multiple independent lineages of STEC have acquired and maintained the same virulence gene repertoire (*36*). Similarly, we have observed this phenomenon with genes of pO113. For example, STEC strains EH43 and EH52, of CG 36 and 31, respectively, possess all 9 of the pO113 genes examined in this study, indicating their independent acquisition of pO113 or a closely related large plasmid.

We determined the genetic makeup of pO113 and highlighted the similarities and differences of pO157. We also demonstrated that LEE-negative STECs are not a clonal group of human pathogens; instead, they encompass a population of evolutionarily distinct STECs that share virulence features but appear to have acquired these features independently and in parallel, rather than from a common ancestor. Therefore, pathogenic STEC may arise when a given set of virulence genes come together in an *E. coli* host. What drives the selection of particular genes to create a STEC pathogen is unknown. However, because the existence of a primarily bovine animal reservoir of infection is a major difference between STEC and other pathotypes of *E. coli*, some genes, such as *ehxA* and *espP*, may be acquired by STEC to facilitate survival and persistence in the bovine gut (*37**,**38*). Therefore, although some determinants may not be considered essential virulence factors for human infection, they may confer an advantage to STEC survival and transmission in a different environment, such as an animal reservoir of infection.

As more plasmid and genome sequences become available, assessing the degree of genetic conservation across LEE-negative serotypes of STEC will be possible. Therefore, persistent public health surveillance and analysis of all STEC associated with human infection is essential to clarify the combination of virulence genes that lead to a STEC pathogen capable of causing serious disease, such as hemorrhagic colitis and HUS. It is critical for public health and clinical laboratories involved in pathogen diagnosis and surveillance to recognize LEE-negative STEC as a cause of human infection.
